# Rapid Cognitive Decline and Recurrent Falls in a 71 Year-Old Man due to Cerebral Amyloidangiopathy-Related Inflammation (CAA-RI)

**DOI:** 10.3390/geriatrics4040056

**Published:** 2019-10-02

**Authors:** Stefan Dörr, Rabea Schickel, Lara Lucke-Paulig, Steffen Schöntag, Ralf Lobmann

**Affiliations:** 1 Department of Endocrinology, Diabetology and Geriatrics, Stuttgart General Hospital, Prießnitzweg 24, 70374 Bad Cannstatt, Germany; r.schickel@klinikum-stuttgart.de (R.S.); r.lobmann@klinikum-stuttgart.de (R.L.); 2 Department of Diagnostic and interventional Radiology, Stuttgart General Hospital, Prießnitzweg 24, 70374 Bad Cannstatt, Germany

**Keywords:** cerebral amyloid angiopathy, CAA, cerebral amyloid angiopathy-related inflammation, CAA-RI, fall in elderly, intracerebral hemorrhage, microbleeding, superficial siderosis, Alzheimer’s disease, amyloid-related imaging abnormalities, ARIA

## Abstract

Cognitive decline and falls in the elderly are common and are often accepted as natural and inevitable by relatives and health care professionals, but frequently there are specific and treatable diseases that should be revealed. In our case, cerebral amyloid angiopathy-related inflammation (CAA-RI) was causative for neuro-psychiatric symptoms and worsening of gait in a 71 year-old man with recurrent falls and decline of gait and cognition. Cerebral amyloidangiopathy (CAA) is an important cause of cerebrovascular disorders in the elderly, characterized by leukoencephalopathy combined with lobar or small cortical hemorrhage due to amyloid deposition in cortical and leptomeningeal vessels. In several conditions, amyloid deposition can provoke inflammation or edema that lead to -normally reversible- encephalopathy. CAA-RI is then characterized by subacute neurobehavioral symptoms, headache, seizures or stroke-like signs. The first therapeutic option after confirming the diagnosis is treatment with glucocorticoids. Despite treatment with prednisolone, our patient could not regain his unrestricted mobility and self-help competence. Our report aims to sharpen awareness for CAA and its inflammatory form (CAA-RI) in healthcare professionals involved in medical care of the elderly and provide a short summary of this disease.

## 1. Case Presentation

### 1.1. Anamnesis

The 71-year-old man was transferred from the emergency ward to our inpatient treatment for the first time after a fall.

He had previously been treated by psychiatrists from April to the middle of June due to progressive cognitive disruption and additional manic symptoms. For example, he spoke about a very important appointment with the German chancellor, and that he will change his hometown into a better place. His daughter was worried and accompanied her father to the psychiatric ward. The psychiatrists diagnosed a manic episode in the course of an affective disorder. An incipient dementia was assumed because of a pronounced leukencephalopathy, a progressed cortical atrophy, and degeneration of brain mass on computer tomography (CT). Beta-amyloid (1–42) in cerebrospinal fluid (CSF) was low (237 pg/mL), beta-amyloid (1–40) was in the normal range (5446 pg/mL), tau-protein was below 100 pg/mL, and phosphor tau-protein was 29.5 pg/mL. The beta-amyloid (1–42)/(1–40)-ratio was low at 4.4%. Thus, an early form of Alzheimer’s disease (AD) could be possible. Because of a sleeping disorder and restlessness, therapy with olanzapine and pipamperone was initiated, and in the absence of any improvement, risperidone was subsequently added. Finally, he was discharged home with the support of his daughter and a nursing service.

Not even one week after discharge from the psychiatric ward, he was admitted to our hospital after a domestic fall with subsequent inability to get up. In this helpless situation, he was found by his daughter, who reported a progressive unsteadiness of his gait and ricketiness in walking over the last week. She also observed a new character change. The patient did not suffer from any chronic somatic diseases and was only taking olanzapine and risperidone since his previous psychiatric treatment. There was no history of anticoagulation, or bleeding or clotting disorders.

### 1.2. Findings

The patient presented with a tendency to fall to the back and right side, instability of the torso, and apraxia. Psychopathologically, he appeared distracted and his short-term memory was reduced, but delusions or hallucinations were not present. Bone fractures were ruled out by radiography. Blood tests did not show any hint of infection, acute kidney failure or electrolyte imbalance, see Table 1.

**Figure 1:**
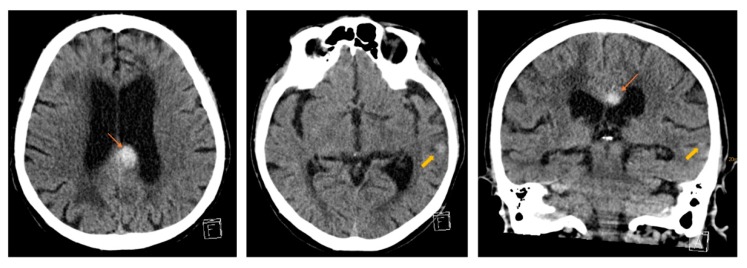
First cranial CT (small arrow—bleeding in the corpus callosum, broad arrow—temporal bleeding).

We performed neuroradiological imaging to rule out ischemic stroke or intracerebral hemorrhage (ICH). We could detect a small temporal subarachnoid hemorrhage and a bleed in the left corpus callosum (see Figure 1). The atypically-located hemorrhage at the corpus callosum was quite suspicious because it might be not fall-related and drew our attention to other causes for ICH, like cerebral amyloid angiopathy (CAA).

**Figure 2:**
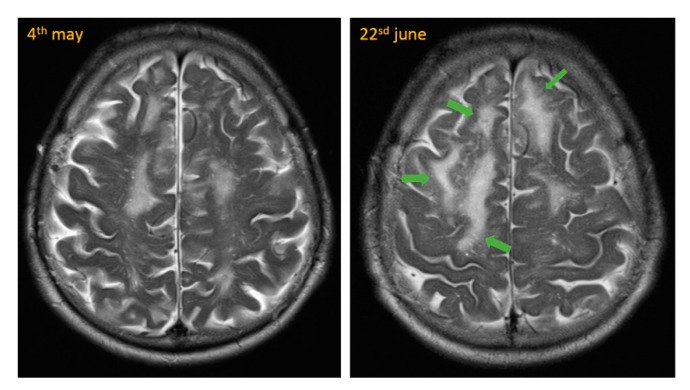
MRI with progressive and new white matter oedema (green arrows) in T2-weighted images.

**Figure 3:**
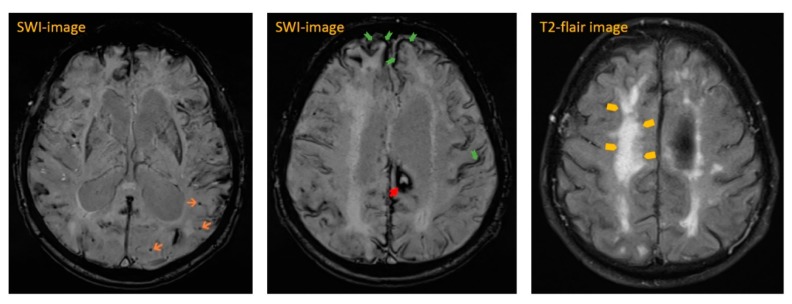
Suspicious findings of CAA-RI in patient’s MRI: micro- (orange colored arrows, left picture) and macrohemorrhage (red arrows, picture in the middle), superficial hemosiderosis (green arrows, picture in the middle) and T2-hyperintensities (yellow arrows, right picture) in susceptibility-weighted images (SWI) and T2-flair images.

The next day, cranial magnetic resonance imaging (MRI) showed multiple cortical-subcortical microhemorrhages and superficial hemosiderosis on susceptibility-weighted images (SWI), which were quite suspicious for CAA (see Figure 2 and Figure 3). In addition, there were white matter hyperintensities (WMH) on T2-weighted images. We were able to compare these images with a study approximately seven weeks prior. Neither progressive white matter hyperintensities nor edema-like changes on MR flair- and T2-weighted sequences were detectable previously. These new changes were highly suspicious for CAA-RI.

The changes in MRI were quite directive, but there were several differential diagnoses, such as cerebral infections and neoplasms, that had to be considered. Therefore, a cerebrospinal fluid (CSF) puncture was executed and it revealed increased total intrathecal protein and leucocytes (see Table 2). Viral and bacterial infections, such as herpes simplex- or JV-virus, or borreliosis, could be excluded. There was no hint of neoplastic growth in the histopathology of the CSF.

Pursuant to the diagnostic criteria for CAA-RI, which were recently published (see Table 3) and which help to distinguish CAA-RI from alternative diagnoses, CAA-RI could be considered as ‘probable’ [1,2,3]. We avoided any effort to get a histopathological biopsy, because of the invasiveness and probable risks of stereotactic surgery. This approach bore the remaining risk of overlooking possible differential diagnoses. However, due to the characteristic changes in behavior, CSF-results and conversion in neuroimages over a temporal course, the diagnosis was confirmed by our neurologists and neuro-radiologists. The elevated erythrocytes in the CSF were seen in the context of ICH.

### 1.3. Differential Diagnosis

Repetitive falls, worsening of gait and change in behavior in the elderly can be provoked by many causes. Hyponatremia and exsiccosis are probably the most common causes in older people, especially when treated with diuretics. Furthermore hyponatremia, exsiccosis and infections can cause delirium, which can appear with similar psychiatric symptoms.

After a fall with inability to get up, ischemic stroke and ICH must consistently be ruled out first, in particular when oral anticoagulation has been taken.

There were several more diagnoses that needed to be considered because the MRI showed atypical hemorrhage, multiple cortical-subcortical microhemorrhages and superficial hemosiderosis in combination with WMH. These alterations were quite suspicious for CAA-RI, but malignant neoplasms are also usually accompanied by steroid-sensitive oedema. Therefore, neoplasms such as brain tumors, meningeosis carcinomatosa and lymphomas should be considered and excluded, as well as central nervous system (CNS) infections.

Aβ-related angiitis (ABRA) is also closely linked to CAA-RI and has a similar clinical presentation, imaging features and response to treatment. There might be a spectrum of CAA-related autoimmune reactions that range from a mere vascular deposit of amyloid β to perivascular inflammation, and finally, transmural vasculitis corresponding to a wide range in clinical severity [4,5].

Since several monoclonal antibodies (mAbs) against amyloid β(Aβ) have risen in the therapy of Alzheimer’s disease (AD), there have been reports about dose-dependent adverse events, especially in ApoE ε4 carriers. Vasogenic edema and microhemorrhage were reported and called amyloid-related imaging abnormalities (ARIA). This represents the major severe side effect of Aβ immunotherapy in AD. In MRI flair-sequences, they appear as vasogenic edema and/or a sulcal effusion (ARIA-E), or as microhemorrhages and superficial siderosis in T2- or susceptibility-weighted images (ARIA-H). Most ARIA cases remain asymptomatic, but they can be associated with non-specific signs and symptoms and a reduction in cognitive performance [6,7].

### 1.4. Therapy

After excluding infectious diseases, we initiated treatment with 1 mg/kg prednisolone based on the diagnosis of a probable CAA-RI. We started with an initial dose of 60 mg daily and reduced it every second day in 5 mg steps. Tapering was planned for 2 weeks. Under this treatment, we recorded improvement of gait and consciousness. His activities of daily living (ADL) improved from 20 to 60, out of 100, points in the Barthel index (BI), and his mobility increased from 10 to 18, out of 28, points in the Tinetti test. An intermittent worsening and rigidity was seen in the context of an extrapyramidal-motoric disorder due to his neuroleptic medication. Risperdal was discarded and replaced by quetiapine and an overlapping tapering of olanzapine. Neck rigidity ameliorated, but he presented with persistent broad-based gait with small steps and cogwheel phenomenon. Finally, he was discharged after 7 weeks inpatient treatment with a daily dose of 30 mg prednisolone (treatment was not started on day one).

### 1.5. Outcome and Follow-Up

**Figure 4:**
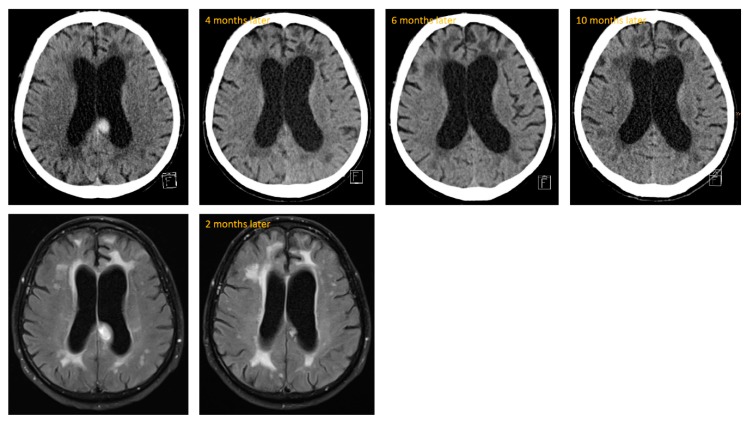
Long-term follow-up on CT (line above) and short-term follow-up on MRI (below).

During outpatient treatment, steroid medication was tapered completely. Two months after discharge from our treatment unit, he was admitted again in a neurology clinic due to worsening of his gait and tendency to fall. Prednisolone was completely tapered at this time and the neuroleptic medication consisted only of olanzapine 5–0–10 mg. The deterioration was thought to be associated with the neuroleptic treatment and advanced CAA. The inflammatory changes of CAA-RI were slightly improved on MRI (see Figure 4). Thus, the neurologists saw no reason to enhance therapy with any other immunosuppressive medications. He also did not receive any further steroid medication. Five days after discharge, he sustained a further fall with a resulting occipital hemorrhage. Following this, he could not regain his complete unrestricted mobility and remained dependent on a domestic 24-h-caregiver. Over the long term, there was no hint of neoplastic growth on CT over the course of about 10 months, but we saw persistent advanced leukencephalopathy (see Figure 4).

## 2. Background Information about Cerebral Amyloid Angiopathy (CAA) and Its Inflammatory Form (CAA-RI)

Cerebral amyloid angiopathy (CAA) is characterized by deposition of amyloid β-protein (Aβ) in vessel walls of cortical or leptomeningeal vessels, typically in small arteries, arterioles and capillaries [8]. These amyloid fibrils trigger progressive deterioration of vascular architecture and alterations such as microaneurysms, vessel occlusion and fibrinoid necrosis. These alterations provoke the distinct symptoms of CAA: coexistence of intracerebral bleeding (micro- and macrohemorrhages), superficial hemosiderosis and ancillary widespread subcortical cerebral leukoencephalopathy [9]. Although usually asymptomatic, CAA can be an important cause of primary lobar intracerebral hemorrhage and contributes to progressive cognitive decline due to hemorrhages and ischemic white matter lesions.

### 2.1. Prevalence

CAA usually occurs sporadically in older individuals, sometimes in association with Alzheimer’s disease (AD), and its appearance is strongly age dependent. Whereas CAA is rarely found in persons younger than 50 years, postmortem studies showed a prevalence of 30% in 60–69-year-old patients, more than 50% in those between 70–89 years and more than 70% in patients older than 90 years [9,10,11]. Familial forms are rare, although the apolipoprotein E (ApoE) ε4 allele is a risk factor for CAA (see below) regardless of concomitant dementia [12].

### 2.2. Pathophysiology: Amyloid β and Apolipoprotein E (ApoE)

The vascular amyloid deposits in the sporadic form of CAA are biochemically quite similar to the material forming the senile plaques in AD (10). How Aβ deposits are initiated and promoted is not well understood. Due to several known mutations in the amyloid precursor protein (APP) gene, susceptibility of the Aβ to its proteolysis or clearance from the central nervous system is decreased and its toxicity toward components of the vessel wall is increased [13].

There is also a relationship between CAA and alleles of apolipoprotein E (ApoE). The most common physiological ApoE in healthy people is ε3. In about two thirds of patients with proven CAA, one or both alleles of ApoE ε2 or ε4 are present. There is evidence that Apo E ε4 facilitates Aβ deposition in patients with CAA or AD. The presence of an ApoE allele is therefore strongly and associated with sporadic CAA in a dose-dependent manner [14,15].

Both alleles are also associated with a higher risk of CAA-related hemorrhage, earlier onset (mean age 75 versus 82 years) and greater risk of hemorrhage recurrence (two-year cumulative recurrence rate of 28% versus 10% for the ApoE ε3/ε3 genotype) [16,17,18].

### 2.3. Cerebral Amyloid Angiopathy-Related Inflammation (CAA-RI)

Cerebral deposits of amyloid are able to provoke inflammation in vessel walls and thus cause white matter edema. This phenomenon was first described in 1974 by Scolding et al. as an isolated diagnostic entity, in contrast to six cases of primary cerebral angiitis of the central nervous system without CAA. Since then, there were several more reports about this “Aβ-related angiits”, currently called “cerebral amyloid angiopathy-related inflammation” (CAA-RI) [19,20].

CAA-RI is a rare type of a potentially reversible encephalopathy in a subset of patients with CAA presenting with acute or subacute cognitive decline rather than hemorrhage. The mean age of affected patients is 67 years, and men and women are equally affected [1]. The course of CAA-RI can be divided into monophasic, remitting or primary progressive forms [2].

The occurrence of CAA-RI is strongly associated with the homozygous frequency of the ApoE ε4 allele, which is found in about 70% of patients with CAA-RI, while it is found in less than 5% of people with CAA without inflammation [1,21,22].

### 2.4. Clinical Signs and Diagnosis of CAA-RI

CAA-RI is characterized by progressive cognitive decline, character changes, neurobehavioral symptoms, focal neurological signs (stroke-like signs), headache and epileptic seizure—symptoms of an acute to subacute encephalopathy [20,23]. Neuroimaging shows potentially reversible leukoencephalopathy consisting of asymmetric patchy or confluent white matter hyperintensities (WMH) on T2-weighted MRI sequences. In validation studies, these changes showed a sensitivity and specificity of 82% and 97%, respectively [3]. As a probable key feature, these white matter lesions often appear as oedema concealing microbleeds in this area.

Recently, diagnostic criteria have been proposed by Chung and Auriel et al., which help to distinguish CAA-RI from alternative diagnoses. The diagnostic criteria are based on clinical appearance and neuroimaging, and allow confirmation of the diagnosis without the need for biopsy (see Table 3). The most important objects are: [1,2,3]

- Age >40 years
- acute to subacute appearance
- headache
- stroke-like symptoms
- neurobehavioral symptoms
- epileptic seizure
- MR-imaging: T2 hyperintense lesions in the cortex and subcortical white matter and CAA-typical changes with microhemorrhages and cortical siderosis.

Cerebrospinal fluid (CSF) may be normal, but often shows a pleocytosis and/or mild raised protein (increased IgG). During the acute phase of inflammation, autoantibodies against Aβ can be detected in CSF but they often return to control levels during remission [19,24,25,26].

To confirm the diagnosis, and especially before starting immunosuppressive therapy, primary CNS infections such as progressive multifocal leukencephalopathy (PML, JC-virus infection), herpes encephalitis, toxoplasmosis, *Cryptococcus* or arthropod transmitted diseases should be excluded.

Viral and autoimmune encephalitis and cerebral neoplasms, e.g., lymphoma or meningeosis carcinomatosa, should always be ruled out by puncture and analysis of CSF, because they can appear similar.

### 2.5. Treatment Options in CAA-RI

Standard treatment in the case of proven CAA-RI is immunosuppression with prednisolone 1 mg per kg weight, which leads to improvement in most cases, although few cases had recurrent symptoms. Other treatment options in the case of a refractory course are methotrexate, mycophenolate-mofetil, cyclophosphamide, immunoglobulins or azathioprine [21,27,28].

Because of the high risk of ICH and the high recurrence rate, anticoagulants and antiplatelet agents should be avoided in patients with CAA. This recommendation is controversial in patients with atrial fibrillation (AF), thus retrospective analyses suggest good outcomes for patients with AF who are restarted on anticoagulation after recovery from an anticoagulant-associated ICH [29].

Statins were also suspected to increase the risk of ICH, because a number of studies showed an inverse relationship between total- and LDL-cholesterol and the risk of ICH [30]. However, treatment with statins appears to neither elevate the risk of primary ICH nor negatively influence the prognosis [31].

## 3. Discussion

Considered retrospectively, the symptoms of our patient were quite characteristic for CAA-RI because they are typically described as character changes, headache, progressive cognitive decline, stroke-like symptoms and/or epileptic seizures. Our patient met the diagnostic criteria of ‘age above 40 years’, ‘acute to subacute appearance’, ‘stroke-like symptoms like torso instability’, ‘neurobehavioral symptoms’ and ‘T2 hyperintense white matter lesions’ as proposed by Chung and Auriel et al. [2]. Furthermore, there were signs of CAA such as microhemorrhages and cortical superficial siderosis on MRI. Perhaps the first admission to the psychiatric clinic was already the first symptom of CAA-RI.

Schaumberg et al. [27], for example, reported seven cases with CAA-RI and five of them suffered preexisting dementia, which became worse within five months or one day—correspondent to a subacute or acute appearance of CAA-RI. These five elderly patients presented with neuro-psychiatric symptoms, such as disturbances of their memory or complex executive functions. They showed problems with walking and talking, dys- or anarthria and, in one case, psychiatric symptoms with delusion and hallucination. Neuro-psychiatric symptoms seem to be predictive for CAA-RI.

Characteristic MR alterations in CAA-RI are reversible, asymmetric, punctual or confluent leukoencephalopathy (white matter hyperintensities, WMH) with emphasis on the frontal cortex [11]. This was also seen in our patient. The early asymmetric, reversible changes can transform to persistent symmetric leukencephalopathy [2]. Our patient also showed persistent leukencephalopathy up to ten months after the primary event (see Figure 4).

Retrospectively, it would have been helpful to estimate anti-Aβ antibodies or the ApoE-genotype in our case. Both could have given an important clue to the diagnosis of CAA-RI, especially in cases when brain biopsy is considered to be harmful. During the acute phase of CAA-RI, anti-Aβ antibodies increase in CSF, together with augmented tau and P-181 tau. Antibodies progressively return to control levels over the course of clinical and radiological remission [25]. Antibodies were unfortunately not tested during the acute phase in our case. There was no hint of infections or neoplastic cells in the patient’s CSF. Neoplastic growth could not be excluded first, because malignant tumors are often accompanied by vasogenic edema and prednisolone therapy ameliorates this edema and tumor growth as well. However, over the long-term, there was no hint of neoplastic growth.

In our case, prednisolone initially improved the situation, but could not restore the patient’s complete physical constitution. Perhaps the functional state, based on vascular leukencephalopathy, had already progressed too far at the time of diagnosis. In addition, neuroleptic treatment may have aggravated his gait.

All patients reported by Schaumberg [27] in 2018 received prednisolone 1 mg per kg weight with an overall good result. Most patients (70–80%) are susceptible to corticosteroids; however, 23 to 25% progress or relapse [1,21]. Of course, before starting immunosuppressive treatment with prednisolone, primary CNS infections must be excluded. In refractory cases, methotrexate (MTX), mycophenolate-mofetil (MMF), cyclophosphamide, immunoglobulins or azathioprine (AZA) are alternative treatment options [21,28]. The patient was assessed as being in too poor a condition to commence immunosuppression with MMF or cyclophosphamide. However, MTX, AZA or immunoglobulins would have been options that should have been discussed. The optimal duration of treatment currently remains unclear and should be adjusted to the course of the disease. Reduction and tapering of the immunosuppression is a case-by-case decision. In our case, the symptoms improved within a few weeks with steroid treatment but then became worse again without a evidence of a relapse, and the patient could not regain his unrestricted mobility. He suffered consistently from falls.

Are there retrospectively things that we should have done different? To forego biopsy of the cerebral lesion was afflicted with uncertainty and could have led to the wrong diagnosis. Therefore, it might have been better to obtain a histopathological diagnosis or at least to examine Aβ antibodies and/or the ApoE genotype. Due to the recurrent falls, it also might have been better to perform MRI once more because CT is not as sensitive as MRI in detecting white matter edema. Perhaps the patient required more intensive immunosuppression.

## 4. Conclusions

This case impressively shows that acute worsening and neuropsychiatric symptoms in the elderly often have a specific cause that should be fathomed. In most cases, exsiccosis, hyponatremia, or acute infection are causative. In this case, the patient’s worsening and fall was caused by CAA-RI. Diagnostic criteria based on clinical and radiological features have been submitted, and therefore, a histological diagnosis is omitted in most individuals, but this bears the risk of misdiagnosis. Assessing the ApoE genotype or Aβ antibodies in CSF during the acute phase are helpful tools to underpin the diagnosis without the necessity of a pathological probe.

Early diagnosis and treatment of CAA-RI can lead to successful therapy, because therapeutic options are available and most cases improve under immunosuppression with prednisolone. Before starting immunosuppressive therapy, bacterial, viral, or prion causes, and neoplastic growth, should be ruled out by CSF puncture. Unfortunately, our patient did not regain his complete mobility under immunosuppressive therapy with steroids caused by progressed vascular leukencephalopathy and an extrapyramidal-motoric disorder due to his neuroleptic medication.

## Figures and Tables

**Table 1:** Blood results of the patient.

Parameter	Normal Range	Result	
Hb	14–18 g/dL	13.8	↓
leukocytes	4–11 Tsd/µL	11.03	↑
creatinine	0.6–1.2 mg/dL	1.0	
eGFR CKD-EPI	60–180 mL/min	75	
Na+	135–145 mmol/mL	137	
K+	3.5–4.5 mmol/L	4.0	
CK	<200 U/L	8706	↑↑
C-reactive protein	<0.5 mg/dL	0.6	↑

↑↑—considerably increased/↑ slightly increased.

**Table 2:** Results of the CSF puncture.

*CSF-results:*	Normal Range	Result	Change
total protein	15–55 mg/dL	68	↑
albumin liquor	<35 g/dL	36.3	↑
albumin l/s-quotient	<8/1000	10.3	↑↑
IgG liquor	<4.0 mg/dL	5.02	↑
IgG l/s-quotient		5.3	
IgA liquor	<0.6 mg/dL	1.73	↑↑
IgA l/s-quotient		5.1	
IgM liquor	<0.1 mg/dL	0.25	↑
IgM l/s-quotient		2.6	
erythrocytes	0/µL	19,000	↑↑
leucocytes	0–5/µL	130	↑↑
neurophiles	<2/µL	120	↑↑
monozytes	<3/µL	10	↑
HSV-PCR	negative	negative	
JCV-PCR	negative	negative	

l/s-quotient—liquor/serum-quotient/HSV—herpes simplex virus/JCV—JC virus. ↑↑—considerably increased/↑ slightly increased.

**Table 3:** Criteria to diagnose CAA-RI [1,3].

Grade of Probability	Criteria
Possible CAA-RI	Age ≥ 40 years. More than one of the following symptoms not directly attributable to an acute ICH: - Headache - impaired consciousness - behavioral change - focal neurological deficit - epileptic seizures MRI: proximate subcortical white matter with WMH. More than one of the following cortico-subcortical hemorrhagic lesions: - cerebral macrobleeds - cerebral microbleeds - cortical superficial siderosis Absence of other infectious or neoplastic causes.
Probable CAA-RI	Age ≥ 40 years. More than one of the following symptoms not directly attributable to an acute ICH: - headache - impaired consciousness - behavioral change - focal neurological deficit - epileptic seizures MRI: asymmetric, uni- or multifocal WMH-lesions in the proximate subcortical white matter. The asymmetric isn’t consequence of ICH. More than one of the following cortico-subcortical hemorrhagic lesions: - cerebral macrobleeds - cerebral microbleeds - cortical superficial siderosis Absence of other infectious or neoplastic causes.
Definite CAA-RI	Criteria of probable CAA-RI plus histopathology findings: Perivascular, transmural and/or intramural inflammation. Proof of amyloid deposits in affected cortex and leptomeningeal regions.

CAA-RI—cerebral amyloid angiopathy-related inflammation/ ICH—intracerebral hemorrhage/WMH—white matter hyperintensity.

## Abbreviations

**Abbreviation**	**Meaning**
AD	Alzheimer’s disease
ApoE	apolipoprotein E
Aβ	amyloid beta
CAA	cerebral amyloid angiopathy
CAA-RI	cerebral amyloid angiopathy-related inflammation
CNS	central nervous system
CSF	cerebrospinal fluid
ICH	intracerebral hemorrhage
mAbs	monoclonal antibodies
SWI	susceptibility-weighted images
